# Case report of 1-stage surgery for a giant arch-descending aortic aneurysm by thoracic endovascular aortic repair under circulatory arrest

**DOI:** 10.1016/j.xjtc.2024.05.017

**Published:** 2024-06-01

**Authors:** Takanori Hishikawa, Takeki Ohashi, Soichiro Kageyama, Akinori Kojima

**Affiliations:** Department of Cardiovascular Surgery, Nagoya Tokushukai General Hospital, Kasugai, Aichi, Japan

**Figure:**
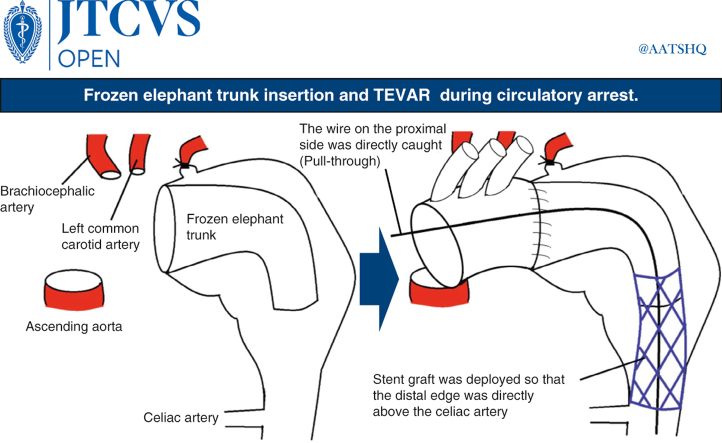

**Figure:**
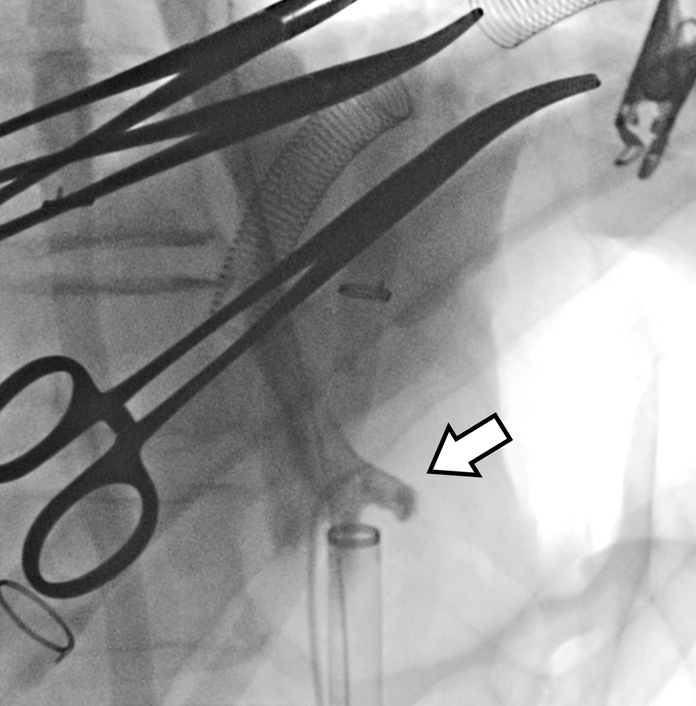
Even during circulatory arrest, the celiac artery could be easily identified by contrast.

**Central Message**

A 1-stage procedure combines a frozen elephant trunk and TEVAR under circulatory arrest for giant aortic aneurysms in a high-risk patient, covering the aneurysm without paraplegia despite risks.

One-stage total thoracic aortic and 2-stage replacements using the elephant trunk technique are both used for aneurysms extending from the ascending and arch aorta to the descending aorta.^1^ Both techniques are associated with high rates of cerebral infarction, paraplegia, and mortality.

A 2-stage surgery with thoracic endovascular aortic repair (TEVAR) is currently the standard procedure. However, in some cases, TEVAR cannot be performed in 2 stages, even if an ascending aortic arch replacement plus an elephant trunk is inserted. This is due to poor atheroma and thrombus in the abdominal and descending aorta from the external iliac artery, posing risk of embolism during catheter operation. Additionally, in cases of impending rupture or rupture at the first stage, emergency surgery is necessary to control bleeding and second-stage TEVAR is not possible. Herein, we present our experience with TEVAR during circulatory arrest of the ascending arch replacement and frozen elephant trunk insertion surgery to minimize the risk of embolism and cover the aortic aneurysm without additional lateral thoracotomy to reduce bleeding.

## Case Presentation

**Figure 1:**
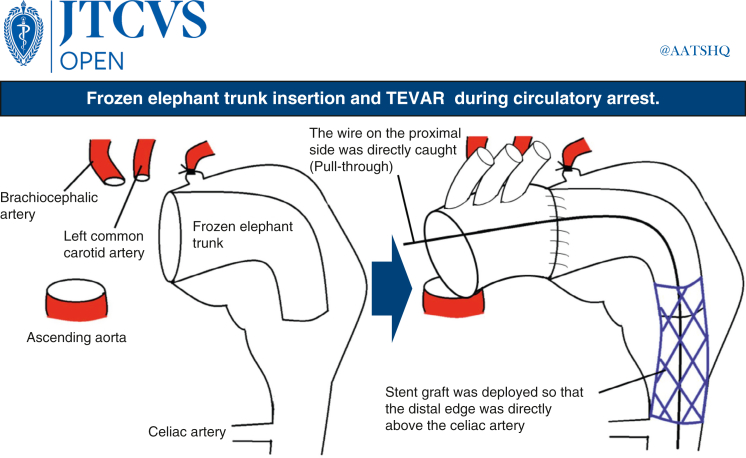
Enlarged mediastinum due to thoracic aortic aneurysm. *TEVAR*, Thoracic endovascular aortic repair.

A 67-year-old man who had retired from a factory job 9 years prior and lived at home alone reported having a cough and shortness of breath on exertion for approximately 2 years. He was unable to go grocery shopping, had lost 18 kg during the past 2 years, and exhibited frailty. A year earlier, he had experienced frequent left back pain and visited a local clinic. A computed tomography scan of the thorax and abdomen revealed a distal arch aortic aneurysm of 142 × 130 mm (Figure 1 and Video 1). No hematoma in the mediastinum or rupture was observed. He did not exhibit hoarseness before surgery. Additionally, no swallowing difficulties during meals attributable to esophageal compression by the thoracic aortic aneurysm were observed. Laboratory test results revealed serum albumin level of 2.1 g/dL, total bilirubin level of 0.6 mg/dL, creatinine level of 0.76 mg/dL, brain natriuretic peptide level of 36.1 pg/mL, and hemoglobin level of 11.5 g/dL. Echocardiography indicated ejection fraction of 70%, left ventricular end-diastolic diameter of 44 mm, and left ventricular end-systolic diameter of 27 mm. No wall motion or significant valvular abnormalities were observed. Preoperative respiratory function evaluation showed vital capacity of the predicted value of 69% and forced expiratory volume in 1 second of 42%, indicating severe respiratory dysfunction.

TEVAR without ventilator use was considered. However, the central landing zone was insufficient and curved and there was a high possibility of an endoleak. The patient was in a state of imminent rupture. Other options considered were aortic artery replacement via the fourth intercostal approach extending from the left midaxillary to the right anterior axillary lines. However, this was considered difficult due to the patient's frailty and the high degree of adhesion to the surrounding lung and esophagus that was expected given the expansive size of the aneurysm and the invasive nature of the surgery. Further, there was a high risk of rupture during the waiting period and systemic embolization due to massive intra-aneurysmal atheroma during TEVAR. Therefore, we performed TEVAR with arch replacement + frozen elephant trunk insertion through a median sternotomy during circulatory arrest to cover the descending aorta in 1 stage and resume circulation, aiming to cover the arch to the descending aorta in 1 stage. Blood was pumped from the ascending aorta, the right atrium was drained to establish an artificial cardiopulmonary system, and the patient was cooled to 25 °C. Firm adhesions were observed in the pericardial sac of the mediastinum and around the aortic arch. The left axillary artery was exposed and anastomosed with a Triplex 8 mm artificial vessel (Terumo). After confirming cooling to 25 °C using a rectal thermometer, the ascending aorta was clamped and myocardial protection fluid was administered progressively. The circulation was stopped and the aorta was cut open.

The aorta was dissected at aortic arch zone 2 and a cerebral isolation circuit was selectively inserted into the brachiocephalic and left common carotid arteries. The left subclavian artery was ligated into the root and a 31 × 90 mm Frozenix (Japan Lifeline) was inserted at zone 2 and anastomosed with a 28-mm artificial vessel with 4 branches (Triplex) using an outer felt. An 8 Fr sheath was inserted into the right common femoral artery and a 6 Fr sheath was inserted into the left common femoral artery. Furthermore, 8 Fr Cobra-shaped catheter (Medikit) and Radifocus (Terumo) were inserted from the 8 Fr sheath. The wire was replaced with Lunderquist (Cook Medical) and the 22 Fr Dryseal (W. L. Gore & Associates, Inc) sheath was replaced. A 7 Fr Atrieve (Cosmotec) snare was inserted into the aneurysm from the central artificial vessel side. A 380-cm radius focus stiff *J*-type snare was inserted from below, caught by the Atrieve snare from above, and pulled through. Cobra-shaped catheter was inserted from the left femoral artery to the celiac artery for contrast. The location of the celiac artery and superior mesenteric artery was determined and the Cobra-shaped catheter was engaged in the celiac artery (Video 2). The Lunderquist wire on the proximal side was directly caught from the artificial vessel, then the TAG (28-28-200) (W. L. Gore & Associates, Inc) was located using the pull-through technique and deployed (Video 3), aligning the distal edge directly above the celiac artery. The TAG (34-34-200) was then deployed (Video 4) so that the proximal edge was located at the central end of the frozen elephant trunk, and TEVAR was completed by touch-up of the whole body using Trilobe (W. L. Gore & Associates, Inc) (Video 5). Blood was pumped through sheaths inserted into the right and left femoral arteries. After blowing off air and debris, circulation was resumed from the side branch of the artificial blood vessel. Circulation was stopped for 86 minutes. The central side of the artificial vessel was anastomosed with the ascending aorta, and the left common carotid artery and brachiocephalic artery were also anastomosed with the side branches of the artificial vessel. The left subclavian artery was anastomosed with a Triplex 8-mm artificial vessel guided into the mediastinum and anastomosed with a side branch of the artificial vessel.

After all anastomoses were completed, fluoroscopic contrast was performed again to confirm the absence of endoleak (Video 6). We also confirmed that the stent graft had not strayed and a partial abdominal branch could be contrasted (Video 7). The heart-lung machine was withdrawn, and the operation was completed. The operation time was 438 minutes, the artificial heart-lung time was 315 minutes, and the cardiac arrest time was 121 minutes. The patient was extubated on postoperative day 2. A contrast-enhanced computed tomography scan was performed on postoperative day 7 to confirm that there was no problem with the artificial vessels (Video 8). No particular complications were observed. However, he continued to experience shortness of breath during the postoperative period. After approximately 1 month, the patient's self-reported symptoms had improved. Respiratory function tests showed improvements in percent vital capacity to 74% and forced expiratory volume in 1 second to 62%. As mentioned earlier, he was weak and found managing daily life alone difficult. His only family member lived far away, which required time to arrange for transfer to their location. Due to the COVID-19 pandemic, hospitals were cautious about accepting transfers, which also took time. The patient was transferred on postoperative day 64 for rehabilitation in preparation for discharge home.

## Discussion

Regarding 1-stage procedure for extensive aortic lesions extending from the ascending and arch aorta to the descending aorta, Kouchoukos and colleagues^2^ performed a fourth intercostal anterior chest opening (clamshell incision) with a sternal transverse incision extending from the left middle axilla to the right anterior axilla lines. In total, 46 cases were reported to have a good hospital mortality rate (6.5%). However, 17% of patients underwent postoperative thoracotomy, 17% required open chest hemostasis, 42% of survivors required ventilator support for more than 72 hours (tracheotomy in 13%), 13% exhibited transient brain damage, and 9% required postoperative dialysis.

In contrast, as a 2-stage treatment, Borst and colleagues^1^ inserted an artificial blood vessel (elephant trunk) into the residual peripheral aortic aneurysm during the first operation through a median sternotomy for a wide thoracic aortic aneurysm. A second operation was widely performed using the elephant trunk for descending aortic replacement through a left open-chest approach.^1^

The 2-stage approach allows for minimally invasive surgery by reducing extracorporeal circulation and circulatory arrest times. The incidence of paraplegia is reported to be extremely low: approximately 1.2% in the first stage and 2% in the second and subsequent stages. Conversely, approximately 9% of patients die while awaiting second-stage surgery, and approximately 40% of patients cannot undergo second-stage surgery for some reason, making performing the procedure on a waiting list problematic.^3^, ^4^, ^5^

In addition, if the aneurysm is chronic, exposing the descending aorta due to adhesions to the surrounding tissues is difficult, which may result in lung damage or injury to the surrounding nerves. Even if minimally invasive second-stage treatment is the goal, each procedure—total arch aortic replacement or descending thoracic aortic replacement—is invasive. Safi and colleagues^4^ reported 8.9% and 7.7% surgical mortality rates, respectively. They also reported a 16% risk of rupture of the residual aneurysm during the waiting period for the second operation.^4^ Therefore, in recent years; TEVAR has been performed as a second-stage treatment to reduce the complications of second-stage surgery. However, TEVAR is not an option when the risk of embolism is high because of atheroma or other defects in the descending aorta. When a rupture or impending rupture occurs, a 2-stage standby procedure becomes impossible.

Performing aortic arch prosthesis replacement and elephant trunk insertion through a median incision and completing TEVAR during circulatory arrest, replacement can be performed from the ascending to the descending aorta in 1 stage, with the following advantages. First, blood can be pumped through the sheath from the femoral artery to blow off the debris in the aorta upon circulation resumption, reducing the risk of peripheral emboli. Second, it can be performed in patients with aortic conditions that preclude TEVAR, such as abundant intravascular atheroma and mural thrombi, while minimizing the risk of embolism. Third, it is effective in patients with ruptured or imminently ruptured aortic aneurysms who require 1-stage coverage of the aneurysm and bleeding control as soon as possible. Fourth, even if there is adhesion between the descending aortic aneurysm and surrounding left lung, the descending aorta can be treated in a single stage. Fifth, skin incisions are limited to midline and bilateral inguinal incisions, which are less invasive. In contrast, performing TEVAR during circulatory arrest prolongs the circulatory arrest time. In our case, TEVAR was performed at a rectal temperature of 25 °C. However, a lower temperature is advisable to prolong the permissible time for circulatory arrest before patients become accustomed to the procedure.

As seen in our case, aspiration pneumonia associated with recurrent nerve dysfunction can be fatal in both frail and older patients.^5^ In this regard, inserting the elephant trunk from the central side of the recurrent nerve, such as in zones 0 to 2, can reduce the risk of postoperative recurrent nerve palsy by eliminating the need for touching the recurrent nerve.

However, there is a significant limitation to this technique: The risk of paraplegia. The risk of paraplegia associated with covering the entire ascending and descending aortas in a single stage should be considered. Due to the high risk of occluding numerous intercostal arteries without reconstruction, spinal cord ischemia may occur. In our case, the absence of paraplegia may be attributed to organ protection achieved under hypothermic conditions at 25 °C, debris removal that could cause embolization by flushing blood flow from the femoral artery before circulation resumed, and the fact that our graft was smaller than the aorta and likely not occluding all the upper-level ostia. Therefore, we suggest that in younger patients without comorbidities such as respiratory impairment, procedures involving rib artery reconstruction, such as left thoracotomy with artificial blood vessel replacement, should be considered as a priority.

Additionally, regarding organ protection in this case, we cooled the patient to 25 °C rectal temperature. The circulatory arrest time was 85 minutes; no organ damage was observed. In this procedure, selectively maintaining perfusion to the abdominal branches during circulatory arrest is impossible; TEVAR must be performed during circulatory arrest as well. Therefore, to protect the organs, cooling to a lower temperature (around 20 °C rectal temperature) might be preferable.

Another factor that makes TEVAR difficult in the absence of circulation is the lack of blood flow. Although central landing is not a problem for the elephant trunk, peripheral landing requires identifying the celiac artery. Even with a weak flow of contrast medium, the position of the partial ventral branch can be adequately confirmed and peripheral landing can be safely performed. Considering more precise positioning, the use of intravascular ultrasound allows for measurement of the branching position of the abdominal aorta and diameter of the aorta, making it effective for determining the location of the stent graft. Additionally, the placement of a small marker catheter in the celiac artery serves as a useful marker for determining stent graft placement. Additionally, in the long term after TEVAR, endoleak could possibly cause blood to re-enter the aneurysm, leading to its expansion. There is also a risk of stent graft migration, which necessitates regular follow-up.

## Conflict of Interest Statement

The authors reported no conflicts of interest.

The *Journal* policy requires editors and reviewers to disclose conflicts of interest and to decline handling or reviewing manuscripts for which they may have a conflict of interest. The editors and reviewers of this article have no conflicts of interest.

## Supplementary Data

Video 1 Contrast-enhanced computed tomography scan of the thoracoabdomen reveals a 142 × 130 mm subacute aortic aneurysm. Video available at: https://www.jtcvs.org/article/S2666-2507(24)00238-4/fulltext.

Video 2 Contrast is used to confirm the location of the celiac artery while the circulation is stopped. Video available at: https://www.jtcvs.org/article/S2666-2507(24)00238-4/fulltext.

Video 3 Despite the presence of aortic tortuosity, the stent graft can still be placed using the pull-through technique. Video available at: https://www.jtcvs.org/article/S2666-2507(24)00238-4/fulltext.

Video 4 Despite the presence of aortic tortuosity, the stent graft can still be placed using the pull-through technique. Video available at: https://www.jtcvs.org/article/S2666-2507(24)00238-4/fulltext.

Video 5 The thoracic endovascular aortic repair procedure is completed by performing touch-up adjustments throughout the entire stent graft using Trilobe. Video available at: https://www.jtcvs.org/article/S2666-2507(24)00238-4/fulltext.

Video 6 Contrast-enhanced computed tomography scan during cardiopulmonary resuscitation to check for endoleaks. No endoleak is observed. Video available at: https://www.jtcvs.org/article/S2666-2507(24)00238-4/fulltext.

Video 7 Contrast-enhanced view of the partial abdominal branch. There is no stent graft occlusion, and the partial abdominal branch is open. Video available at: https://www.jtcvs.org/article/S2666-2507(24)00238-4/fulltext.

Video 8 Postoperative contrast computed tomography scan showing thrombosis in the mass. Video available at: https://www.jtcvs.org/article/S2666-2507(24)00238-4/fulltext.
