# Supplementary figures and images for: Case report of 1-stage surgery for a giant arch-descending aortic aneurysm by thoracic endovascular aortic repair under circulatory arrest
Source: JTCVS Tech. 2024 Jun 1;26:10–5. doi: 10.1016/j.xjtc.2024.05.017 (PMC11329206; doi:10.1016/j.xjtc.2024.05.017)

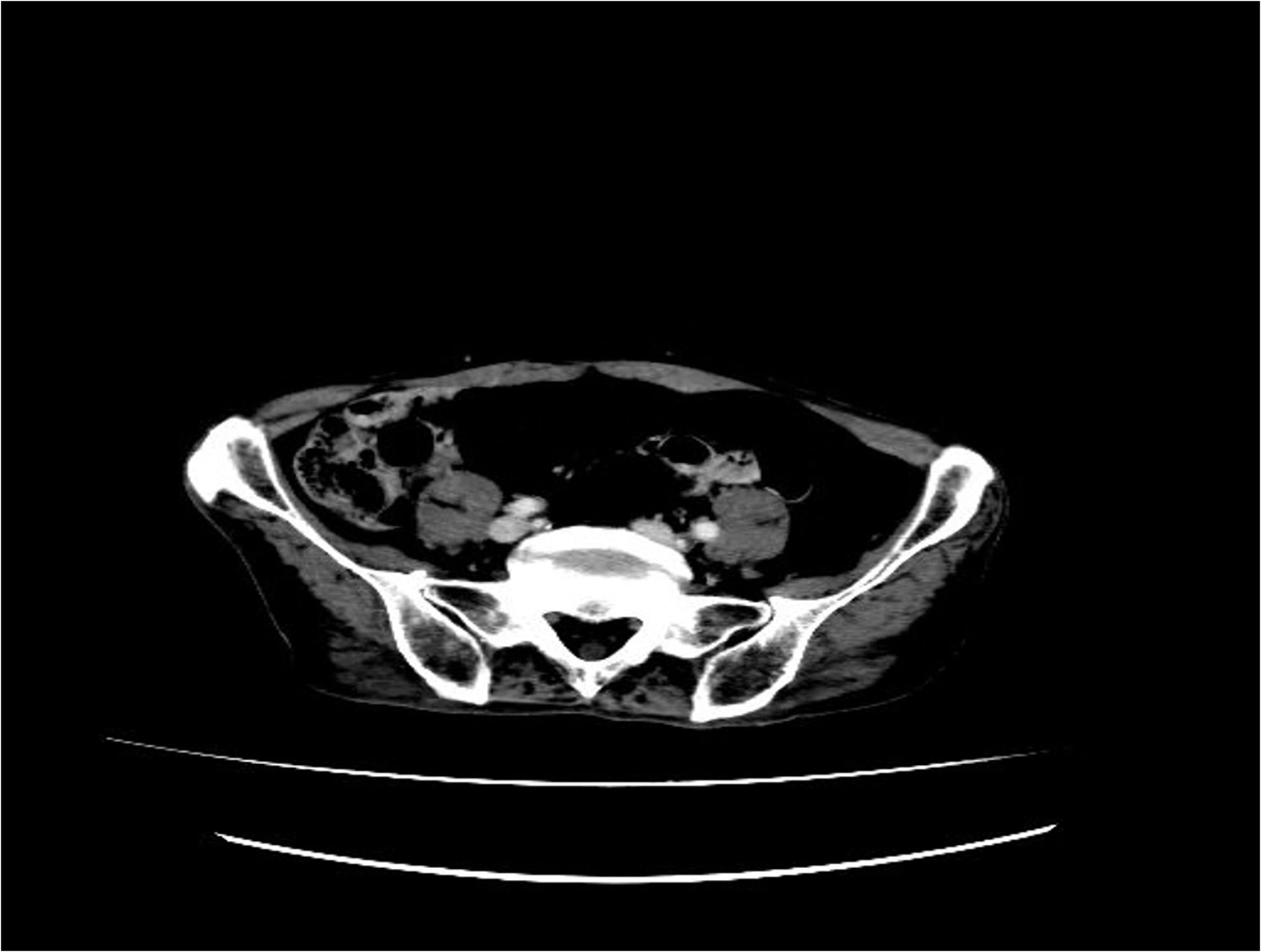

Supplement: Video 1 — Contrast-enhanced computed tomography scan of the thoracoabdomen reveals a 142 × 130 mm subacute aortic aneurysm. Video available at: https://www.jtcvs.org/article/S2666-2507(24)00238-4/fulltext. [file fx2.jpg]

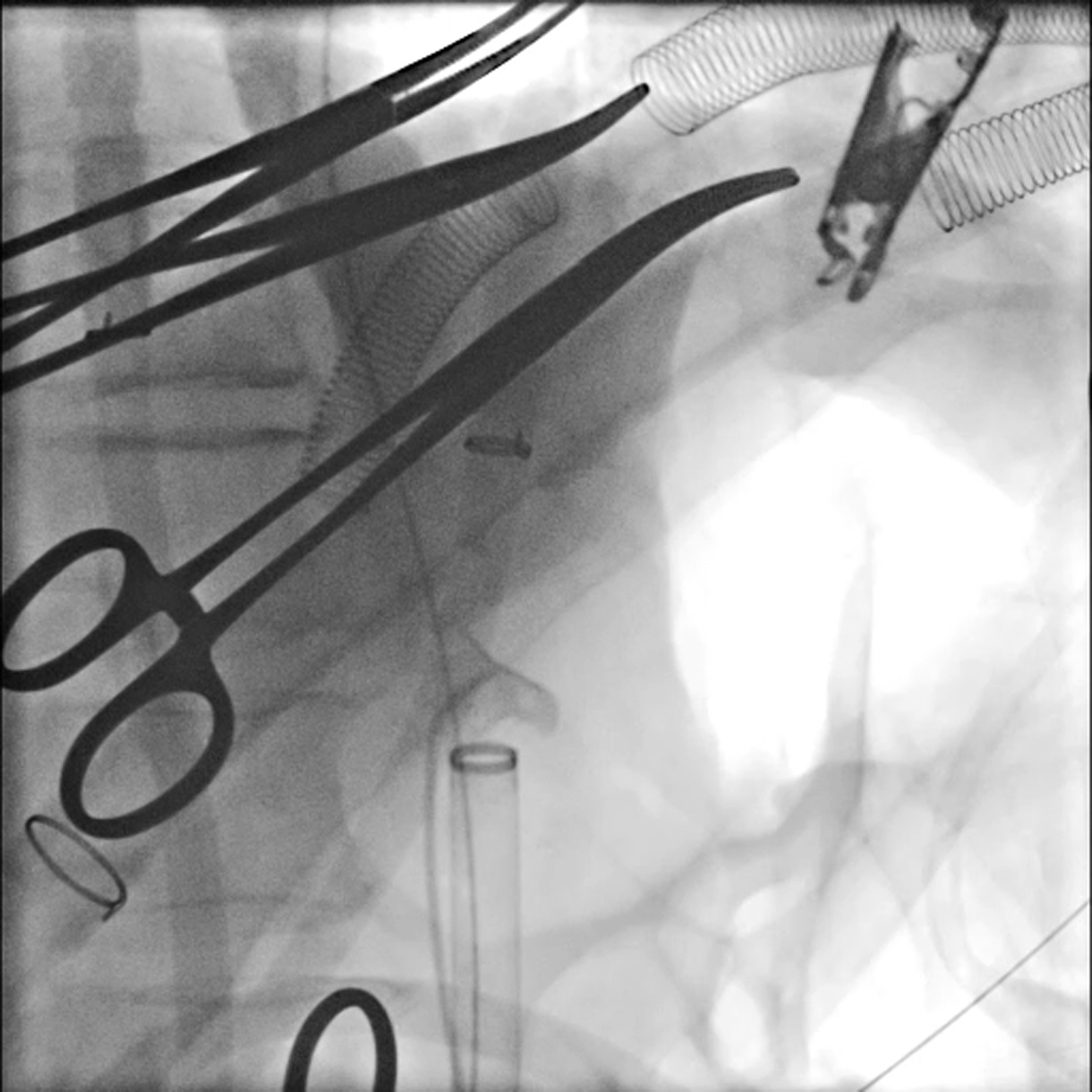

Supplement: Video 2 — Contrast is used to confirm the location of the celiac artery while the circulation is stopped. Video available at: https://www.jtcvs.org/article/S2666-2507(24)00238-4/fulltext. [file fx3.jpg]

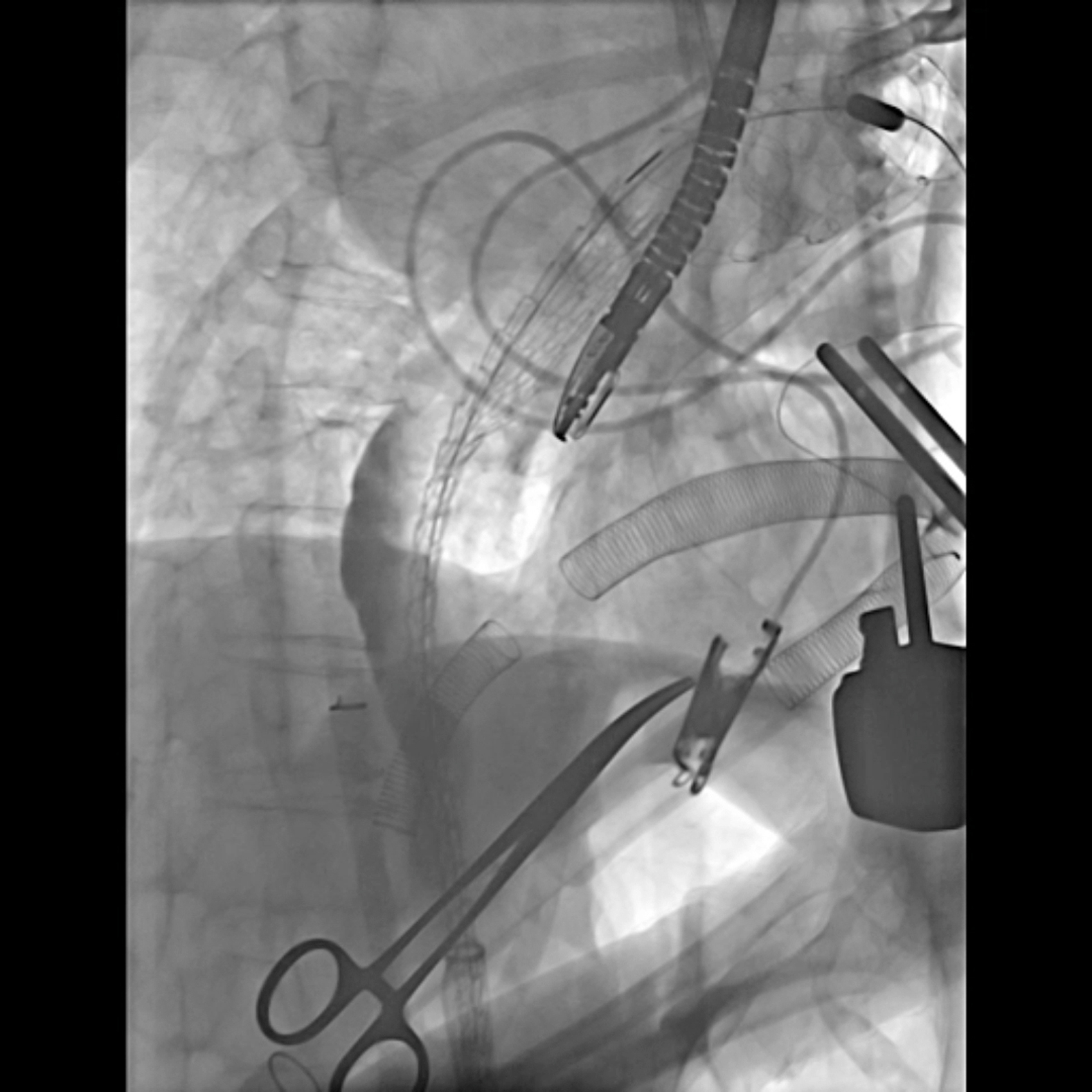

Supplement: Video 3 — Despite the presence of aortic tortuosity, the stent graft can still be placed using the pull-through technique. Video available at: https://www.jtcvs.org/article/S2666-2507(24)00238-4/fulltext. [file fx4.jpg]

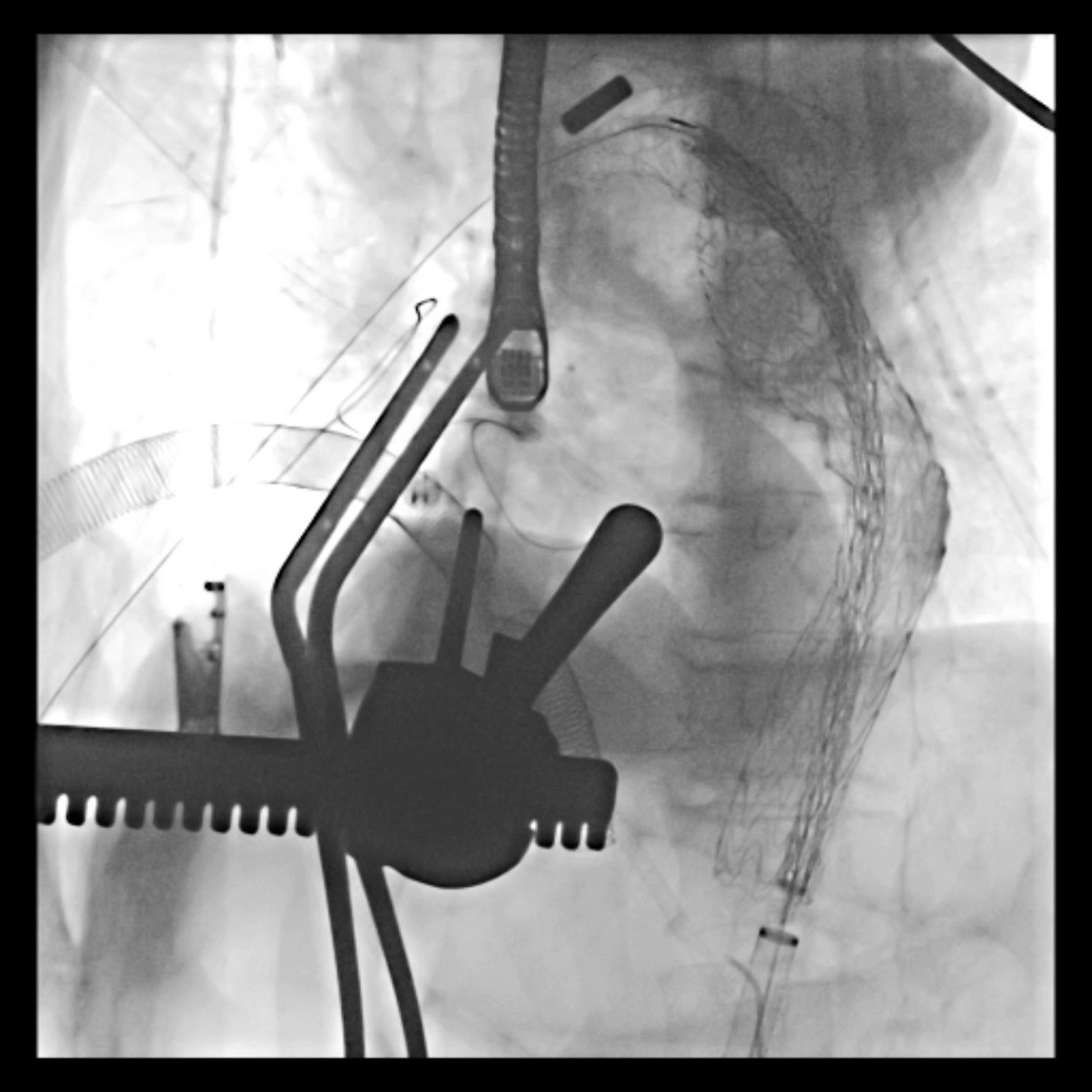

Supplement: Video 4 — Despite the presence of aortic tortuosity, the stent graft can still be placed using the pull-through technique. Video available at: https://www.jtcvs.org/article/S2666-2507(24)00238-4/fulltext. [file fx5.jpg]

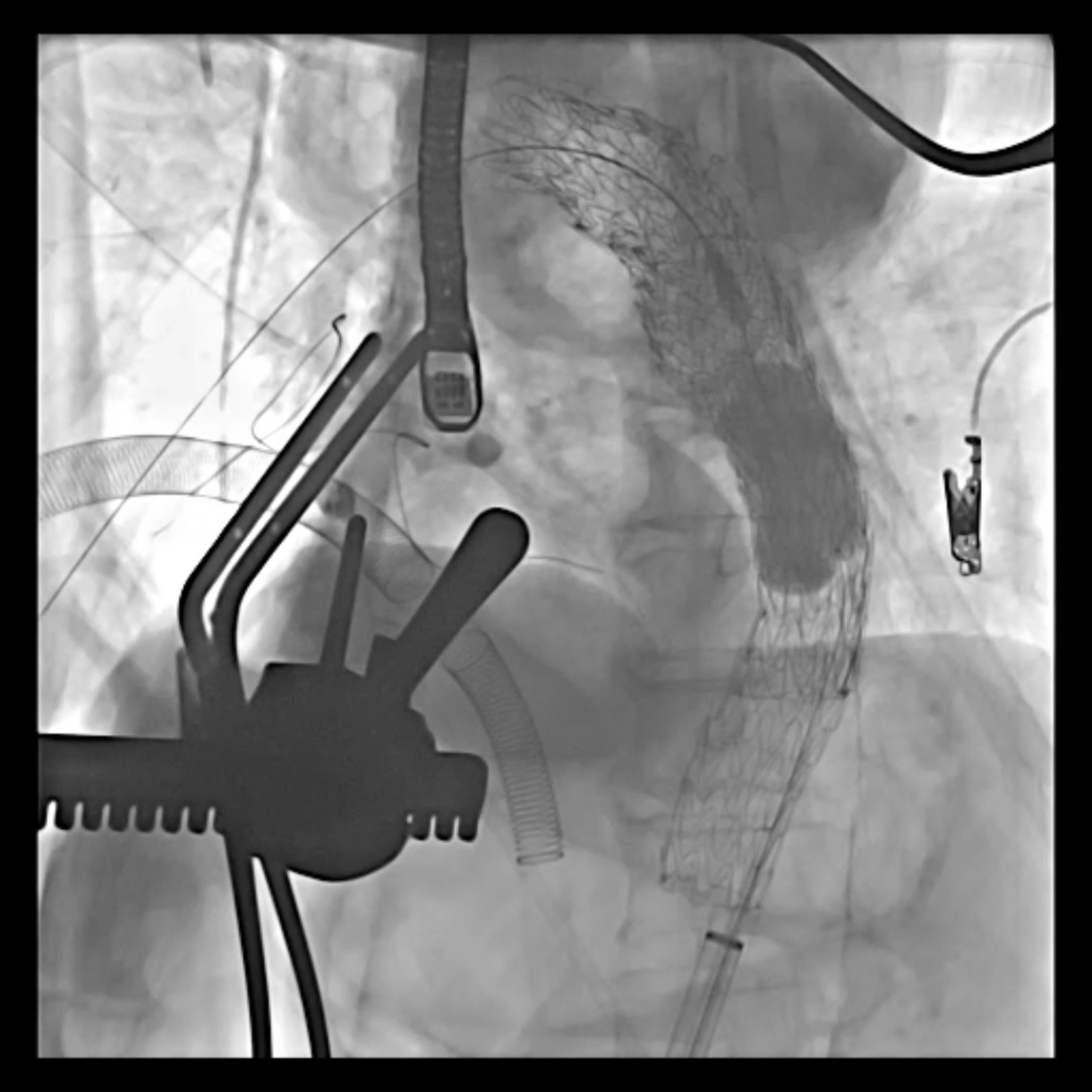

Supplement: Video 5 — The thoracic endovascular aortic repair procedure is completed by performing touch-up adjustments throughout the entire stent graft using Trilobe. Video available at: https://www.jtcvs.org/article/S2666-2507(24)00238-4/fulltext. [file fx6.jpg]

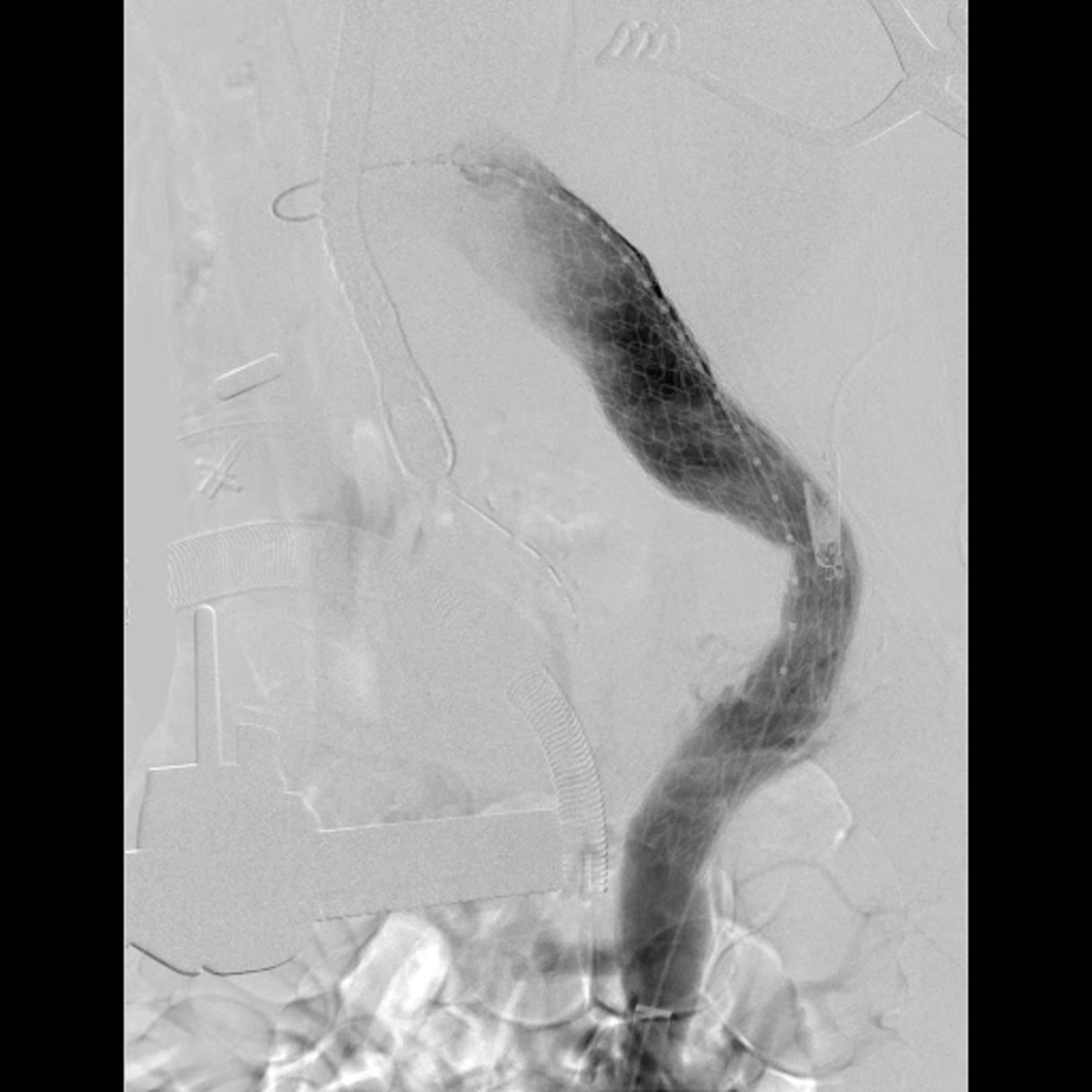

Supplement: Video 6 — Contrast-enhanced computed tomography scan during cardiopulmonary resuscitation to check for endoleaks. No endoleak is observed. Video available at: https://www.jtcvs.org/article/S2666-2507(24)00238-4/fulltext. [file fx7.jpg]

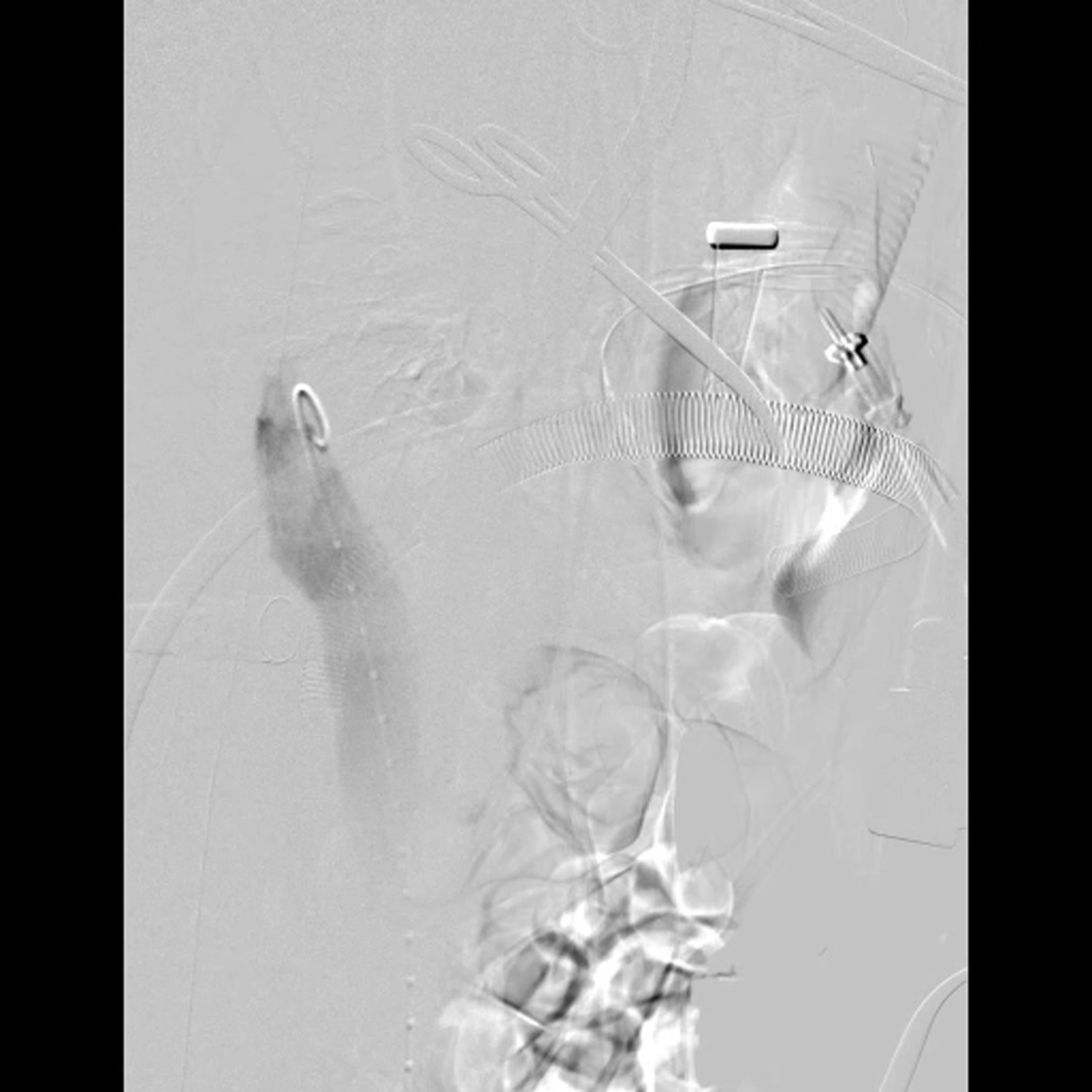

Supplement: Video 7 — Contrast-enhanced view of the partial abdominal branch. There is no stent graft occlusion, and the partial abdominal branch is open. Video available at: https://www.jtcvs.org/article/S2666-2507(24)00238-4/fulltext. [file fx8.jpg]

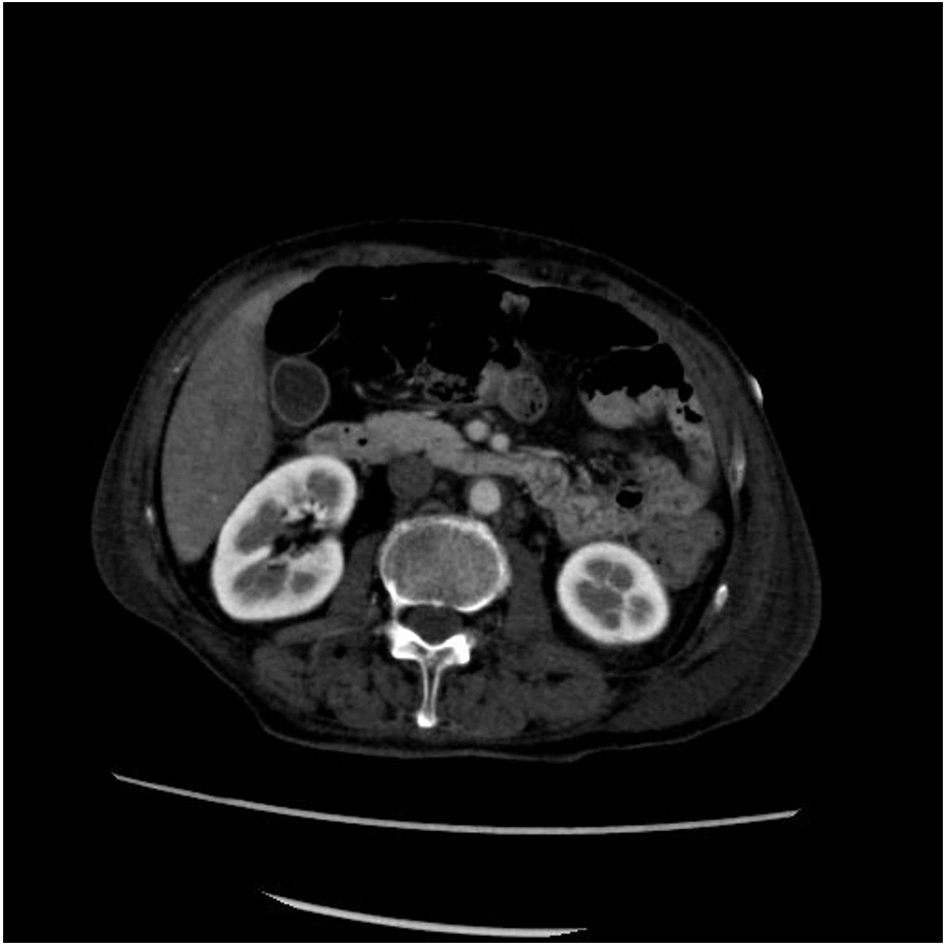

Supplement: Video 8 — Postoperative contrast computed tomography scan showing thrombosis in the mass. Video available at: https://www.jtcvs.org/article/S2666-2507(24)00238-4/fulltext. [file fx9.jpg]
